# Influence of Cycloplegia on Axial Length Prediction Models in a Paediatric Sample

**DOI:** 10.1007/s44402-026-00095-3

**Published:** 2026-05-18

**Authors:** Ivo Soares, António Baptista, Oscar Torrado, Pedro Serra

**Affiliations:** 1 Department of Physics, University of Beira Interior, Covilhã, Portugal; 2 Health Sciences Research Centre (CICS-UBI), University of Beira Interior, Covilhã, Portugal; 3 Clinical and Experimental Centre in Vision Sciences (CCECV), University of Beira Interior, Covilhã, Portugal; 4 Physics Centre of Minho and Porto Universities (CF-UM-UP), Braga, Portugal; 5 Ophthalmology Clinic Vista Sánchez Trancón, Badajoz, Spain

**Keywords:** Axial length, Cycloplegia, Myopia, Paediatric, Prediction models, Refractive error

## Abstract

**Clinical Relevance:**

Accurate axial length (AL) estimation is vital for monitoring myopia progression in children, especially in primary care where optical biometers are often unavailable. Prediction models with cycloplegic measurements may offer a reliable alternative.

**Purpose:**

To assess the effect of cycloplegia on the accuracy and repeatability of several AL prediction models in a paediatric sample and to identify which models maintain minimal bias under both cycloplegic and non-cycloplegic conditions.

**Methods:**

Ninety-six children (mean age 12.5 ± 2.4 years) underwent repeated measurements of spherical equivalent refraction (SER), anterior corneal curvature (*K*_mean_) and AL, pre- and post-cycloplegia, using the Myopia Master. Seven published prediction models incorporating SER, *K*_mean_, age and sex were evaluated. Agreement, bias, limits of agreement (LoA), coefficient of repeatability (CoR), intraclass correlation coefficient (ICC) and regression analyses were used to assess performance and repeatability.

**Results:**

Cycloplegia induced a hyperopic shift (mean +0.79 D), most pronounced in emmetropic and hyperopic eyes. Measured AL and all models showed improved repeatability post-cycloplegia (measured AL CoR decreased from ~0.14 mm to ~0.09 mm; ICC > 0.99). Pre-cycloplegia, models overestimated AL (mean differences from –0.87 to –0.24 mm); these biases were reduced post-cycloplegia (mean differences from –0.56 to +0.10 mm). Models by Morgan et al., Queirós et al. and Lingham had the smallest bias (<0.10 mm) and narrowest LoA (<0.84 mm). Variation in SER accounted for ~97–99% of the change in predicted AL, while the *K*_mean_ contributed ≤1.2%.

**Conclusion:**

Cycloplegic refraction significantly enhanced both accuracy and repeatability of AL prediction models in children. Models by Morgan et al., Queirós et al. and Lingham et al. performed best. Predictive models may be a valuable substitute in settings without access to optical biometers, provided cycloplegic measurements are used when possible.

Key Points

- The cycloplegic refraction substantially improves both the accuracy and repeatability of axial length prediction models in myopic, emmetropic and hyperopic children.
- The spherical equivalent is the main factor influencing the accuracy of axial length prediction models, while changes in corneal curvature have only minimal impact.
- Axial length prediction models provide a useful approach for estimating axial length in children when optical biometers are unavailable; however, their utility in myopia progression management may be limited.

## Introduction

Myopia is a rapidly increasing global ocular condition, largely attributed to prolonged engagement in near-work activities [1], reduced outdoor exposure [2] and genetic predisposition [3]. Its escalating prevalence represents a major public health concern, not only because of more individuals requiring refractive correction and frequent ophthalmic care, but also due to the lifelong risk of sight-threatening pathologies associated with myopia [4]. These complications can ultimately lead to severe visual loss and reduced quality of life [5, 6],

Myopia progression is intrinsically linked to the axial elongation of the eye [7], which strongly correlates with visual impairment [4]. Consequently, axial length (AL) measurement is a key biometric parameter for monitoring refractive development [8]. The ratio of AL to anterior corneal radius also correlates strongly with spherical equivalent refractive error (SER) [9] and may serve as an indicator for detecting myopia onset [10].

AL can be measured using ultrasound or optical coherence biometers [11]. While ultrasound requires corneal contact and local anaesthesia, optical biometers—based on interferometric or coherence-based technologies—offer superior precision and accuracy, achieving approximately ±0.1 mm (~0.25 D), even in paediatric populations [12]. Despite their utility, particularly in cataract surgery planning, optical biometers are available mainly in tertiary centres. In contrast, myopia detection and monitoring typically occur in primary care settings where such devices are less accessible [13].

To address the lack of direct AL measurement in primary care, several studies have developed theoretical [14] and statistical [13, 15–21] models to predict AL using routinely obtained parameters. Common predictors include SER and corneal curvature (keratometry), with some models incorporating demographic or biometric variables such as age, sex [16, 17, 20], weight [19] and anterior chamber depth [21]. However, differences in population characteristics, measurement devices and testing conditions (e.g., cycloplegic vs. non-cycloplegic refraction) can affect model accuracy and agreement with measured AL. Among these predictors, SER is particularly sensitive to accommodation. When measured without cycloplegia, accommodative effort induces a transient myopic shift, underestimating hyperopia and overestimating myopia, which may lead to overestimated AL predictions [22–24].

Morgan et al. reported only a marginal difference in the limits of agreement when comparing predictions of AL based on cycloplegic (±0.73 mm) and non-cycloplegic (±0.75 mm) data [13], while Tang et al. reported a decrease in the difference between predicted and measured AL as well as an improvement in the range of agreement [16].

To our knowledge, no previous study has evaluated these predictive models within the same population. The present study assessed the accuracy of multiple AL prediction models under cycloplegic and non-cycloplegic conditions in a shared paediatric sample. Additionally, it examines the intrasession repeatability of these models when refraction and keratometry are measured sequentially.

## Methods

### AL Prediction Model Selection

A comprehensive literature search was conducted in PubMed, Web of Science, Scopus and Cochrane databases for studies published between 2000 and 2025 on AL prediction formulas. Models were selected if they used input variables routinely measured in clinical practice — SER, keratometry, age and sex. The search strategy combined terms related to AL, prediction or modelling and clinical parameters. Only models based on optical coherence interferometry were included. The selected prediction models are summarised in Table 1.

**Table 1:** Formulas for predicting axial length using the input variables spherical equivalent refraction (SER), mean keratometry (*K*_mean_), Sex or Age.

First author	Model
He** [15] (2015)	${{AL}}_{{HE}}=\left(\frac{{K}_{{mean}}}{10.724}\right)* \left({SER}-32.208+0.234* {Sex}\right)$
Kim* [14] (2019)	${{AL}}_{{Kim}}=24.00* \left(\frac{{K}_{{mean}}}{7.80}\right)-\left(0.40* {SER}\right)$
Tang** [16] (2020)	${{AL}}_{{Tang}}=40.31+\left(0.056* {Age}\right)-\left(0.013* {Sex}\right)-\left(0.396* {K}_{{mean}}\right)-\left(0.353* {SER}\right)$
Morgan**[13] (2020)	$\displaystyle{{AL}}_{{Morgan}}=\frac{1}{\frac{0.22273}{{K}_{{meam}}}+\left(0.00070* {SER}\right)+0.01368}$
Queirós* [17] (2022)	*AL*_Queirós_ $=\left(0.019* {Age}\right)+\left(2.271* {K}_{{mean}}\right)-\left(0.444* {SER}\right)+5.414$
Dutt** [18] (2022)	${{AL}}_{{Dutt}}=\left(2.102* {K}_{{mean}}\right)-\left(0.415* {SER}\right)+7.268$
Lingham** [20] (2024)	${{AL}}_{{Lingham}}=5.472-\left(0.069* {Age}\right)+\left(3.060* {\log }_{10}\left({Age}\right)\right)-\left(0.265* {Sex}\right)+\left(0.135* {SER}\right)+\left(2.019* {K}_{{mean}}\right)-\left(0.072* {SER}* {K}_{{mean}}\right)$

*Non-cycloplegic SER, **Cycloplegic SER.

### Participants

This secondary analysis used data from a previously reported prospective cross-sectional study (single-visit; no follow-up) and the same sample of 96 paediatric participants [25]. That investigation evaluated the repeatability and agreement of Myopia Master (version 7.2 R3; Oculus, oculus.de/us) measurements under cycloplegic and non-cycloplegic conditions. The present work addresses a distinct question — the evaluation of AL prediction models using this dataset. The original measurement procedures, including the cycloplegia protocol, measurement sequence, fixation target and quality control criteria, followed the validated methodology reported by Peñaranda et al. [25] and are summarised below.

The 96 participants (48 females, 50%; mean age 12.5 ± 2.4 years, range 7–16 years) underwent a comprehensive ophthalmological examination at Clínica Oftalmológica Vista Sánchez-Trancón (Spain). Inclusion criteria were refractive astigmatism <2.50 D under cycloplegia, distance-corrected visual acuity of 6/6 or better and the absence of ocular pathology or strabismus. All measurements were performed under ophthalmological supervision.

The examination protocol included assessments of distance visual acuity (with and without correction), objective autorefraction (with and without cycloplegia), anterior corneal curvature measurements, AL measurement, subjective refraction, cover test, slit-lamp biomicroscopy and fundus examination.

Autorefraction, keratometry and AL were measured using the Myopia Master in ‘Myopia Mode’. The device is a closed autorefractometer/biometer that incorporates a fixation target simulating optical infinity with a fogging system to control accommodation. Two measurements (average of three measurements each) were performed automatically and sequentially, following the manufacturer’s protocol. Only measurements meeting predefined quality thresholds were included (quality index ≥7 for autorefraction and keratometry; signal-to-noise ratio ≥6.0 for AL).

Before cycloplegia, autorefraction, anterior corneal curvature and AL were each measured twice within a 5-min interval to assess intrasession repeatability. Cycloplegia was induced with one drop of cyclopentolate (1% cyclopentolate, Alcon, alcon.com), followed by a second drop after 10 min. Post-cycloplegia measurements were performed 30 min after the initial instillation and were repeated twice within 5 min. All measurements were performed by the same experienced optometrist to ensure procedural consistency.

The SER was calculated as sphere + (cylinder/2) and mean anterior corneal curvature (*K*_mean_) as the average of the flat and steep meridians. The study adhered to the Declaration of Helsinki, was approved by the independent regional ethics committee (Comité Ético para Investigación Clínica de Badajoz), and written informed consent was obtained from parents or legal guardians.

### Data and Statistical Analysis

The unit of analysis was the participant; although measurements were performed on both eyes in this dataset, inferential analyses used only one eye per participant (right eye for all participants) to avoid non-independence of fellow-eye data. All statistical analyses were performed using JupyterLab (v4.0.11; Project Jupiter, jupyter.org). Descriptive statistics are reported as mean ± standard deviation (M ± SD), together with range and 95% confidence interval (95% CI), the same for the mean difference. Data normality was assessed using the Kolmogorov–Smirnov test. Participants were classified as myopic (SER ≤ –0.50 D), emmetropic (–0.50 D < SER < +2.00 D) or hyperopic (SER ≥ +2.00 D) based on the cycloplegic SER [26].

### Statistical Power Calculation

The study had 88% power to detect a mean difference <0.2 mm between the measured and predicted AL, assuming a standard deviation of 0.5 mm and a Bonferroni-adjusted significance level of 0.007 (0.05/7). A difference of 0.2 mm corresponds to ≈0.50D, which is considered clinically relevant in the management of paediatric myopia.

### Repeatability Analysis

Intrasession repeatability of AL predictions relies on SER and *K*_mean_ measurements. Directly measured values and the model-predicted values were evaluated using three complementary metrics. First, the within-subject standard deviation (Sw) quantified the variability between repeated measurements for each participant. Second, the coefficient of repeatability (CoR) was calculated as 2.77× Sw, with its 95% CI estimated from the chi-squared distribution. This coefficient represents the smallest detectable change that can be interpreted as exceeding the expected measurement noise under identical conditions. Third, the intraclass correlation coefficient (ICC) was computed using a two-way random-effects model for absolute agreement [ICC(2,1)], accounting for variability attributable to both participants and repeated measures. ICC was interpreted as follows: <0.50, poor; 0.50–0.75, moderate; 0.75–0.90, good and >0.90, excellent [27]. This approach is particularly suitable for assessing the reliability of repeated measurements obtained with the same device under consistent testing conditions.

Differences between repeated measurements were analysed using two-tailed paired *t*-tests. To avoid type I errors arising from multiple comparisons across the seven predictive models and the measured AL, the significance threshold was adjusted using Bonferroni correction (*α* = 0.05/8 = 0.006).

### Agreement Analysis

Agreement between measured and model-predicted AL was evaluated under both cycloplegic and non-cycloplegic conditions. For each participant, the reference AL was defined as the mean of two repeated measurements. Predicted AL values were derived using each model formula, with SER and *K*_mean_ obtained from the same measurement condition.

Agreement was assessed through Bland–Altman analyses and quantified by the mean difference between measured and predicted AL values, representing systematic bias. The 95% limits of agreement (LoA) were defined as the mean difference ±1.96 times the SD of the paired differences, indicating the interval within which 95% of the differences are expected to lie. Confidence intervals for the LoA were also computed to reflect estimate precision.

The intraclass correlation coefficient [ICC(3,1)] was used to assess relative reliability, i.e., the consistency with which measured and predicted AL values rank across participants, irrespective of systematic bias. The coefficient of variation within subjects (CV_WS_), expressed as a percentage, was calculated as the within-subject SD divided by the mean AL. Together, these metrics provided a scale-independent assessment of prediction reliability.

Paired *t*-tests were used to detect systematic bias between predicted and measured AL. Significance was again adjusted using the Bonferroni correction (*α* = 0.05/7 = 0.007).

### Influence of Input Variation on Model-Predicted AL

The effect of variability in pre-cycloplegic inputs on predicted AL was examined using multiple linear regression, with post-cycloplegic measurements serving as the reference standard. The dependent variable was the difference between pre- and post-cycloplegic predicted AL (ΔAL). Independent variables included changes in SER (ΔSER) and mean corneal curvature (Δ*K*_mean_), while static factors (e.g., age, sex) were excluded. These ΔALs were then correlated with the corresponding differences in SER (ΔSER) and *K*_mean_ (ΔK_mean_), to determine the extent to which variability in each parameter contributed to deviations in model-predicted AL relative to its cycloplegic baseline.

## Ethics Approval

This study was carried out in accordance with the principles of the Declaration of Helsinki and was approved by the independent regional ethics committee (Comité Ético para Investigación Clínica de Badajoz).

## Results

The cohort comprised 38 myopes, SER = −2.48 ± 1.24 D (range: −5.64 to −0.79  D), 41 emmetropes, SER = +0.71 ± 0.58 D (range: −0.42 to +1.95 D) and 17 hyperopes, SER = +3.43 ± 1.53 D (range: +2.10 to +7.25 D) – see Table 2.

**Table 2:** Demographic, refractive and biometric data pre- and post-cycloplegia, represented by mean ± standard deviation and range.

		Pre-cycloplegia* M ± SD (range)			Post-cycloplegia* M ± SD (range)		
	*n*	SER(D)	*K*_mean_(mm)	AL (mm)	SER(D)	*K*_mean_(mm)	AL(mm)
Myopes	38	−2.71 ± 1.30 (−6.27 to −0.72)	7.77 ± 0.25 (7.20 to 8.34)	24.40 ± 0.97 (22.05 to 26.68)	−2.33 ± 1.29 (−5.64 to −0.54)	7.78 ± 0.25 (7.19 to 8.37)	24.40 ± 0.96 (22.05 to 26.70)
Emmetropes	41	−0.05 ± 0.59^‡^ (−1.54 to 1.15)	7.77 ± 0.29 (7.27 to 8.47)	23.06 ± 0.75 (21.49 to 24.40)	0.71 ± 0.58^‡^ (−0.42 to 1.95)	7.77 ± 0.29 (7.29 to 8.49)	23.09 ± 0.77 (21.48 to 24.46)
Hyperopes	17	1.65 ± 1.65^‡^ (−0.52 to 5.84)	7.86 ± 0.30 (7.11 to 8.24)	22.05 ± 0.82 (20.43 to 23.21)	3.43 ± 1.53^‡^ (2.10 to 7.25)	7.86 ± 0.30 (7.11 to 8.26)	22.04 ± 0.83 (20.41 to 23.22)
All	96	−0.80 ± 2.01^†^ (−6.27 to 5.84)	7.79 ± 0.28 (7.11 to 8.47)	23.41 ± 1.22 (20.43 to 26.68)	−0.01 ± 2.38^†^ (−5.64 to 7.25)	7.79 ± 0.28 (7.11 to 8.49)	23.42 ± 1.23 (20.41 to 26.70)

*Average of two measurements; *AL* axial length; *D* dioptres,F, *K*_mean_ Mean anterior corneal curvature, *M* Mean, *SD* standard deviation, *SER* spherical equivalent, ^†^Paired *t*-test; *p* = 0.01, ^‡^Paired *t*-test; *p* < 0.001.

Comparison of pre- and post-cycloplegic data showed statistically significant SER shifts toward more hyperopic refractions in emmetropes, hyperopes and the combined group. Mean differences for emmetropes, hyperopes and the overall sample were −0.76 ± 0.60 D (95% CI: −0.83 to 0.43 *p* < 0.001), −1.79 ± 1.21 D (95% CI: −2.41 to −1.16, *p* < 0.001) and −0.79 ± 0.32 D (95% CI: 0.16 to 1.42, *p* = 0.01), respectively. Myopes showed no significant difference pre- and post-cycloplegia (mean difference = −0.38 ± 0.25 D; 95% CI: −0.98 to 0.22; *p* = 0.20). Anterior corneal curvature and AL differences were not statistically significant (*p* > 0.20).

### Intrasession Repeatability of Measured and Predicted AL

Table 3 shows that the mean difference between consecutive AL measurements and predicted AL derived from consecutive SER and *K*_mean_ was close to zero, both pre- and post-cycloplegia. Measured AL demonstrated the weakest CoR pre-cycloplegia (0.14 mm, 95% CI: 0.13 to 0.17 mm) and post-cycloplegia (0.09 mm, 95% CI: 0.08 to 0.11 mm), outperforming all AL predictive models. Among the models, Tang et al. achieved the best repeatability pre-cycloplegia (0.24 mm, 95% CI: 0.21 to 0.28 mm) and post-cycloplegia (0.16 mm, 95% CI: 0.14 to 0.18), whereas that of He et al. showed the poorest pre-cycloplegia (0.48 mm, 95% CI: 0.42 to 0.56 mm) and post-cycloplegia (0.27 mm, 95% CI: 0.24 to 0.32 mm). Overall, both measured and predicted AL improved in CoR repeatability after cycloplegia. Intraclass correlations for measured and predicted AL were excellent under both conditions (ICC > 0.99, with a range for 95% LoA of 0.98 to 1.00).

**Table 3:** Intrasession repeatability of measured and predicted AL.

Pre-cycloplegia				
Method	Measurement 1 M ± SD (mm)	Measurement 2 M ± SD (mm)	Mean difference ± SD (mm) (95% CI)	CoR (mm) (95% CI)
Measured AL	23.41 ± 1.22	23.41 ± 1.21	−0.00 ± 0.07 (−0.02 to 0.01)	0.14 (0.13 to 0.17)
He et al. [15]	23.88 ± 1.67	23.89 ± 1.65	−0.01 ± 0.24 (−0.06 to 0.04)	0.48 (0.42 to 0.56)
Kim et al. [14]	24.28 ± 1.11	24.29 ± 1.11	−0.01 ± 0.14 (−0.04 to 0.02)	0.28 (0.25 to 0.33)
Tang et al. [16]	24.11 ± 0.91	24.11 ± 0.91	−0.01 ± 0.12 (−0.03 to 0.02)	0.24 (0.21 to 0.28)
Morgan et al. [13]	23.65 ± 0.95	23.66 ± 0.95	−0.01 ± 0.13 (−0.03 to 0.02)	0.25 (0.22 to 0.29)
Queirós et al. [17]	23.69 ± 1.05	23.70 ± 1.04	−0.01 ± 0.15 (−0.04 to 0.03)	0.30 (0.26 to 0.35)
Dutt et al. [18]	23.97 ± 0.97	23.97 ± 0.96	−0.01 ± 0.14 (−0.03 to 0.02)	0.27 (0.24 to 0.32)
Lingham et al. [20]	23.87 ± 1.06	23.88 ± 1.05	−0.01 ± 0.14 (−0.04 to 0.02)	0.28 (0.25 to 0.33)

Post-cycloplegia				
Method	Measurement 1 M ± SD (mm)	Measurement 2 M ± SD (mm)	Mean difference ± SD (mm) (95% CI)	CoR (mm) (95% CI)
Measured AL	23.42 ± 1.22	23.42 ± 1.22	0.00 ± 0.05 (−0.01 to 0.01)	0.09 (0.08 to 0.11)
He et al. [15]	23.32 ± 1.86	23.32 ± 1.87	−0.01 ± 0.14 (−0.04 to 0.02)	0.27 (0.24 to 0.32)
Kim et al. [14]	23.97 ± 1.20	23.98 ± 1.21	−0.01 ± 0.10 (−0.03 to 0.01)	0.20 (0.17 to 0.23)
Tang et al. [16]	23.83 ± 1.00	23.84 ± 1.01	−0.01 ± 0.08 (−0.02 to 0.01)	0.16 (0.14 to 0.18)
Morgan et al. [13]	23.36 ± 1.02	23.37 ± 1.03	−0.01 ± 0.08 (−0.02 to 0.01)	0.16 (0.14 to 0.18)
Queirós et al. [17]	23.35 ± 1.17	23.36 ± 1.18	−0.01 ± 0.09 (−0.02 to 0.01)	0.18 (0.160 to 0.21)
Dutt et al. [18]	23.65 ± 1.08	23.65 ± 1.09	−0.01 ± 0.09 (−0.02 to 0.01)	0.17 (0.15 to 0.20)
Lingham et al. [20]	23.54 ± 1.16	23.54 ± 1.17	−0.01 ± 0.09 (−0.02 to 0.01)	0.17 (0.15 to 0.20)

*AL* axial length, *CI* confidence interval, *CoR* coefficient of repeatability, *M* mean, *SD* standard deviation. Mean differences were not statistically significant in any of the cases (all *p* > 0.49).

### Agreement Between Measured and Predicted AL

**Fig. 1:**
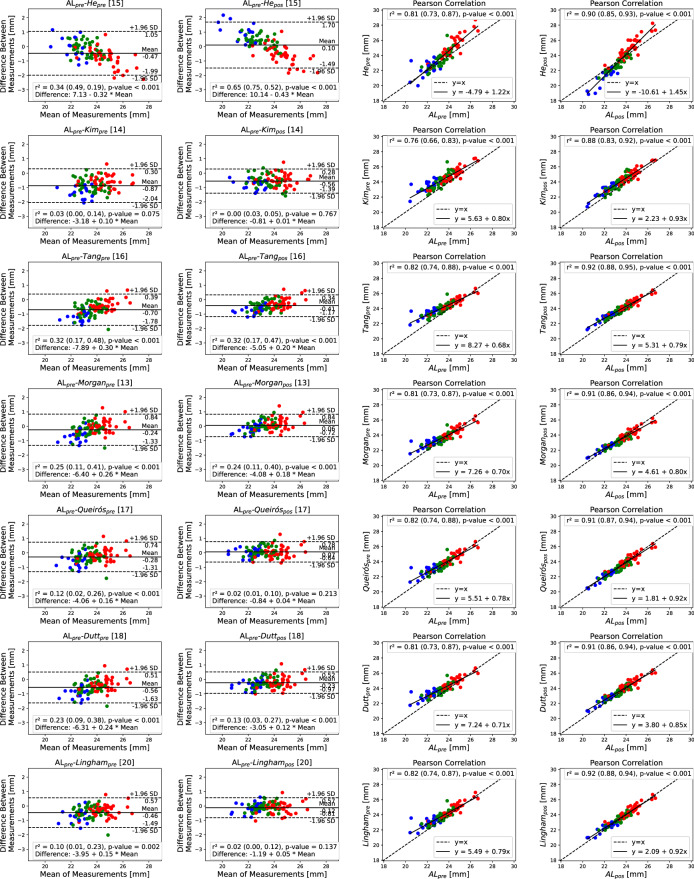
Analysis of axial length (AL) prediction against measured AL (*n* = 96). Red, green and blue dots indicate myopic, emmetropic and hyperopic participants, respectively. The first and second columns shows the Bland−Altman plots for pre- and post-cycloplegia, respectively. The black solid line represents the mean difference between the measured and predicted values (measured – predicted). The black dashed lines are the 95% limits of agreement. The third and fourth columns shows the Pearson correlation analysis of predicted and measured AL values for pre- and post-cycloplegia, respectively. The solid black lines represent the regression model and the dashed line the identity line (*y* = *x*). Pre-cycloplegia Bland–Altman plots show systematic overestimation of AL with wide limits. Post-cycloplegia, bias approaches zero with narrow limits, indicating improved prediction. Regression plots show reduced deviation from unity, with model differences (e.g., Kim [14] vs. Queirós [17]) still evident.

Pre−cycloplegia, all predictive models overpredicted measured AL significantly (*p* < 0.001 for all; Table 4 and Fig. 1). The largest bias was observed with the Kim et al. model [14] (mean difference = −0.87 ± 0.60 mm, 95% CI: −0.99 to –0.75 mm) and the smallest with the predictive model of Morgan et al. [13] (mean difference = −0.24 ± 0.55 mm, 95% CI: −0.35 to –0.13 mm). Kim et al. [14] also showed the widest LoA (−2.05 to +0.30 mm) and the highest CV_ws_ (3.13%), whereas Queirós et al. [17] exhibited the narrowest LoA (−1.31 to −0.74 mm) and the lowest CV_ws_ (1.78%). Intraclass correlations indicated good−to−excellent reliability (all ICC≧0.81).

**Table 4:** Agreement between measured and estimated AL pre- and post-cycloplegia.

Pre-cycloplegia							
Prediction model	AL Predicted	AL Measured	Mean difference±SD (mm) (95% CI)	Lower LoA (mm) (95% CI)	Upper LoA (mm) (95% CI)	CV_WS_^‡^ (%)	ICC^§^ (95%LoA)
	M ± SD (mm)						
He et al. [15]	23.88 ± 1.65	23.41 ± 1.21	−0.47 ± 0.78 * (−0.63 to –0.31)	−1.99 (−2.26 to −1.81)	1.05 (0.87 to 1.32)	2.70	0.90 (0.76 to 0.95)
Kim et al. [14]	24.28 ± 1.11		−0.87 ± 0.60 * (−0.99 to –0.75)	−2.05 (−2.25 to –1.90)	0.30 (0.16 to 0.51)	3.13	0.81 (−0.12 to 0.94)
Tang et al. [16]	24.11 ± 0.90		−0.70 ± 0.55 * (−0.81 to –0.59)	−1.78 (−1.96 to −1.65)	0.39 (0.25 to 0.56)	2.64	0.84 (0.02 to 0.95)
Morgan et al. [13]	23.65 ± 0.95	23.41 ± 1.21	−0.24 ± 0.55 * (−0.35 to –0.13)	−1.33 (−1.50 to –1.19)	0.84 (0.71 to 1.02)	1.81	0.92 (0.85 to 0.95)
Queirós et al. [17]	23.69 ± 1.04		−0.28 ± 0.52 * (−0.39 to −0.18)	−1.31 (−1.47 to –1.18)	0.74 (0.62 to 0.91)	1.78	0.93 (0.85 to 0.96)
Dutt et al. [18]	23.96 ± 0.96		−0.56 ± 0.55* (−0.67 to –0.45)	−1.63 (−1.82 to –1.59)	0.51 (0.39 to 0.70)	2.32	0.88 (0.34 to 0.95)
Lingham et al. [20]	23.87 ± 1.05		−0.46 ± 0.52* (−0.57 to –0.35)	−1.49 (−1.65 to –1.36)	0.57 (0.44 to 0.73)	2.08	0.91 (0.59 to 0.96)

Pre-cycloplegia							
Prediction model	AL Predicted	AL Measured	Mean difference±SD (mm) (95% CI)	Lower LoA (mm) (95% CI)	Upper LoA (mm) (95% CI)	CV_WS_^‡^ (%)	ICC^§^ (95%LoA)
	M ± SD (mm)						
He et al. [15]	23.32 ± 1.86	23.42 ± 1.22	0.10 ± 0.81^†^ (−0.06 to +0.26)	−1.49 (−1.76 to −1.30)	1.69 (1.50 to 1.96)	2.47	0.93(0.89 to 0.95)
Kim et al. [14]	23.98 ± 1.21		−0.56 ± 0.43* (−0.65 to −0.47)	−1.40 (−1.55 to −1.30)	0.28 (0.18 to 0.43)	2.09	0.92 (0.22 to 0.98)
Tang et al. [16]	23.42 ± 1.22		−0.41 ± 0.38* (−0.49 to –0.33)	−1.15 (−1.28 to –1.07)	0.33 (0.25 to 0.46)	1.68	0.94 (0.53 to 0.98)
Morgan et al. [13]	23.36 ± 1.03	23.42 ± 1.22	0.06 ± 0.40^†^ (−0.02 to 0.14)	−0.72 (−0.86 to –0.63)	0.84 (0.75 to 0.98)	1.21	0.97 (0.95 to 0.98)
Queirós et al. [17]	23.35 ± 1.17		0.07 ± 0.36^†^ (0.0 to 0.14)	−0.64 (−0.76 to –0.55)	0.78 (0.69 to 0.90)	1.11	0.98 (0.96 to 0.98)
Dutt et al. [18]	23.65 ± 1.08		−0.23 ± 0.38* (−0.31 to −0.15)	−0.89 (−1.10 to –0.97)	0.51 (0.43 to 0.64)	1.33	0.96 (0.91 to 0.98)
Lingham et al. [20]	23.54 ± 1.17		−0.12 ± 0.35* (−0.19 to −0.05)	−0.81 (−0.92 to –0.72)	0.57 (0.48 to 0.68)	1.12	0.98 (0.96 to 0,98)

*AL* axial length; CI – Confidence Intervals; CV_WS_ - coefficient of variation; ICC- Intraclass correlation coefficients; LoA – Limits of Agreement; M ± SD - Mean ± standard deviation; SD - Standard deviation of differences. *Statistically significant at *p* < 0.007 (Bonferroni−adjusted significance level). ^†^ *p* > 0.05. ^§^Two-way random-effects model.

Post-cycloplegia, mean differences between predicted and measured AL decreased across all models. Kim et al. [14] retained the largest bias (mean difference = –0.56 ± 0.43 mm, 95% CI: –0.65 to –0.47 mm), while Morgan et al. [13] showed the smallest (mean difference = 0.06 ± 0.40 mm, 95% CI: –0.02 to 0.14 mm). He et al. [15] had the widest LoA (–1.49 to 1.69 mm) and highest CV_ws_ (2.47%), while Lingham et al. [20] demonstrated the narrowest LoA (–0.81 to 0.57 mm) and one of the lowest CV_ws_ (1.12%). Reliability improved further (ICC ≥ 0.92; 95% LoA: –0.12 to 0.94 mm), post-cycloplegia.

A consistent proportional bias was observed: shorter eyes (hyperopic and emmetropic) were overpredicted, whereas myopic eyes showed more accurate estimates. The exception was the He et al. model [15], in which hyperopic eyes were underpredicted, emmetropic eyes showed near-accurate estimates and myopic eyes were overpredicted.

**Fig. 2:**
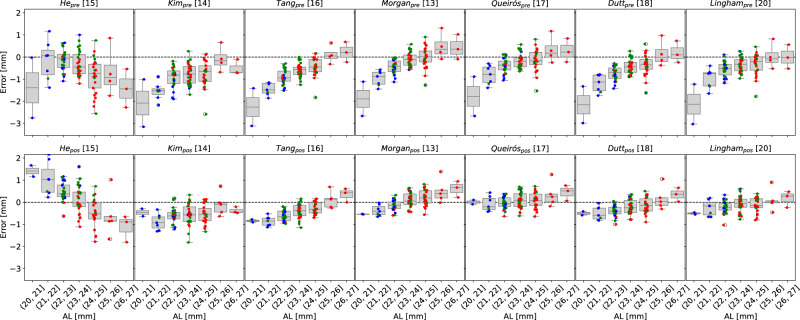
Estimated axial length (AL) error for each predictive model both pre- (top row) and post-cycloplegia (bottom row) for several AL intervals (negative error values indicate model overestimation). Red, green and blue dots indicate myopic, emmetropic and hyperopic participants, respectively.

**Fig. 3:**
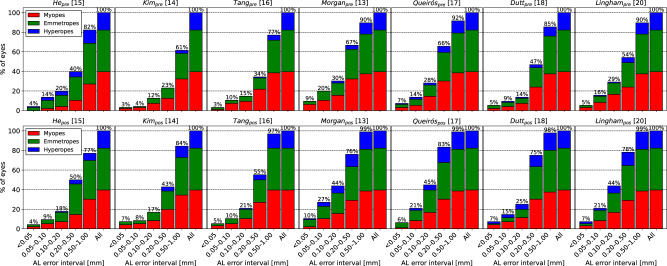
Percentage of eyes with axial length (AL) prediction below threshold, pre-cycloplegia (top row) and post-cycloplegia (bottom row) for the models tested.

Grouped AL analysis confirmed that cycloplegia reduced the overestimation in hyperopic and emmetropic eyes, while in general, the underestimation was maintained in myopes (Fig. 2). Post-cycloplegia, the proportion of eyes with prediction errors <0.5 mm increased, from 23% to 43% for Kim et al. [14] and from 66% to 83% for Queirós et al. [17], indicating lower prediction errors. Post-cycloplegia, the Tang et al. [16], Morgan et al. [13], Queirós et al. [17] and Lingham et al. [20] models predicted AL within ±1.00 mm in >97% of eyes, (Fig. 3).

### Influence of SER and *K*_mean_ on Cycloplegic Associated Variations in Model-Predicted AL

**Fig. 4:**
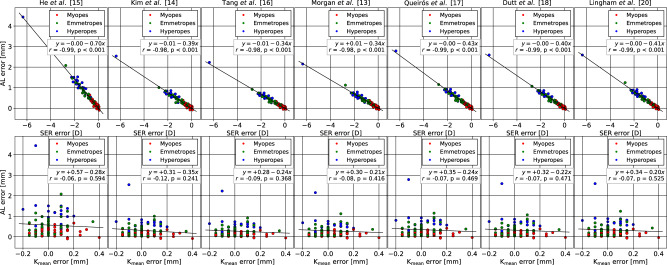
Relationship between spherical equivalent refraction (SER) measurement error (ΔSER = pre - post) and axial length (AL) prediction error (ΔAL = pre - post) (top row) and between the mean keratometry (*K*_mean_) measurement error (Δ*K*_mean_ = pre- post) and ΔAL (bottom row) for the models tested.

Figure 4 illustrates the relationship between cycloplegia-induced changes in spherical equivalent (ΔSER) and mean corneal curvature (Δ*K*_mean_) with variation in predicted AL (ΔAL). Across all models, ΔSER— defined as post- minus pre-cycloplegic SER —was the main driver of ΔAL variation, whereas Δ*K*_mean_ had minimal impact. Multivariate linear regression (Table 5) confirmed this trend, with all models being statistically significant, explaining 98.8–99.7% of the variance in ΔAL. The contribution of ΔSER accounted for 97.3–99.1% of the explained variance, while Δ*K*_mean_ contributed only 0.5–1.2%. These findings demonstrate that cycloplegic-induced changes in SER are the principal determinant of variation in predicted AL, whereas alterations in corneal curvature play a negligible role.

**Table 5:** Multivariate linear regression parameters for axial length (AL) prediction error (ΔAL = AL pre-cycloplegia – post-cycloplegia), respectively, for the models tested.

Model	*β*_1_	*β*_2_	Error	Adjusted *R*²	*p*-value
He et al. [15]	−0.997	−0.073	0.036	0.996	<0.001
Kim et al. [14]	−0.989	−0.139	0.030	0.992	<0.001
Tang et al. [16]	−0.994	−0.111	0.020	0.995	<0.001
Morgan et al. [13]	−0.990	−0.102	0.033	0.987	<0.001
Queirós et al. [17]	−0.996	−0.093	0.022	0.996	<0.001
Dutt et al. [18]	−0.996	−0.093	0.020	0.996	<0.001
Lingham et al. [20]	−0.995	−0.084	0.027	0.994	<0.001

β_1_ and β_2_ are the regression coefficients of ΔSER (pre-cycloplegia – post-cycloplegia); Δ*K*_mean_ (pre-cycloplegia - post-cycloplegia).

*K*_mean_ mean keratometry, SER, spherical equivalent refractive error.

## Discussion

This study compared the performance of several AL prediction models in a paediatric population, both before and after cycloplegia. Overall, the best AL prediction models were Morgan et al. [13], Queirós et al. [17] and Lingham et al. [20], with proven low bias and excellent post-cycloplegic repeatability. All models evaluated were based on linear regression incorporating ocular (SER and *K*_mean_) and demographic (age and sex) parameters, with SER showing a strong association with AL [9]. It was hypothesised that variability in SER measurements could affect model performance. The findings indicate that, across all models, AL prediction accuracy and repeatability improved following cycloplegia, with four of the seven models showing LoA for AL prediction within 1.0 mm of the measured values after cycloplegia. These were the Morgan et al. [13] (LoA: −0.72 to 0.84), Queirós et al.(LoA: −0.64 to 0.78), Dutt et al. [18] (LoA: −0.84 to 0.51) and Lingham et al. [20] (LoA; −0.81 to 0.57) models.

Cycloplegia shifted the measured refractive error towards increasingly positive values (+0.79 D), with the effect being more pronounced in emmetropes (+0.76 D) and hyperopes (+1.79 D) [25]. This refractive shift is consistent with values reported by Hu et al. (+0.78 ± 0.79 D) in children aged 4–18 years [28] and large-scale studies of 12-year olds (+0.84 D) [22]. Additionally, Sankaridurg et al. showed that under non-cycloplegic conditions, the measured refractive error tends to be more myopic (paired difference: −0.63 ± 0.65 D). Larger differences were observed in younger and hyperopic eyes, reflecting a greater accommodative effort [24]. In line with these findings, Figs. 1 and 2 demonstrate that larger AL prediction errors occur in hyperopic eyes, likely due to the greater accommodation required to bring the fixation target into focus [23]. Differences in *K*_mean_ and AL remained within the limits of repeatability of the instrument, indicating that these anatomical parameters were unaffected by cycloplegia [25, 29].

Before cycloplegia, all models overpredicted AL, with mean differences ranging from –0.87 mm (Kim et al. [14]) to –0.24 mm (Morgan et al. [13]), and LoA extending up to 1.18 mm from the mean. In the He et al. [15] model, LoA were even broader, spanning 1.53 mm. After cycloplegia, the prediction errors decreased: the Kim et al. [14] model overpredicted by –0.56 mm, whereas the He et al. [15] model underpredicted only slightly (+0.10 mm). The models proposed by Morgan et al. [13] and Queirós et al. [17] showed mean differences <0.10 mm, corresponding to refractive errors <0.25 D based on the Gullstrand model eye. The LoA also narrowed (<0.84 mm) for all models except He et al. [15], whose precision remained similar to pre-cycloplegic conditions. Using the relationship of 0.4 mm axial elongation per 1.0 D [30], these LoA still represent potential differences of about 2.0 D —highlighting that AL estimations from predictive models should be interpreted cautiously.

Bland–Altman and regression analyses (Fig. 1) show that most models (except He et al. [15]) tended to overpredict AL in emmetropic and hyperopic eyes and underpredict in myopic eyes. Grouping eyes by AL range (Fig. 2) revealed that prediction errors were greatest in shorter eyes (i.e., hyperopic and emmetropic) and reduced post-cycloplegia. Myopic eyes showed smaller, more stable errors. Post-cycloplegia, especially for the Queirós et al. [17] and Lingham et al. [20] models, 50% of eyes with an AL between 21 and 26 mm clustered around the zero-error line, with 95% of cases within 1.0 mm. The observed reduction in proportional bias post-cycloplegia appears particularly relevant in shorter eyes, representative of hyperopia.

The performance of a prediction model depends on its developmental methodology, the population used and the independent variables selected. These factors influence the model’s generalisability. All models reviewed share a strong dependence of AL on SER and anterior corneal curvature, grounded in the principles of ocular refraction. He, Tang, Queirós and Lingham et al. [15–17, 20] further incorporated age and sex, which improved model fit and are known to influence AL.

He et al. demonstrated that ~83% of SER variance was explained by the AL/ *K*_mean_ ratio [15]. They developed an SER prediction model based on AL, *K*_mean_ and sex, later reformulated here to predict AL. Their sample comprised 3922 Asian children aged 6–12 years with cycloplegic refraction. The model showed underprediction in hyperopes and overprediction in myopes—opposite to other models—likely because their SER model estimated more positive SER in hyperopes and more negative SER in myopes. Sex-related differences in AL (males: +0.44 mm) and *K*_mean_ (females: +0.13 mm) were consistent with the present data (AL: +0.70 mm; *K*_mean_: +0.19 mm, data not shown), suggesting that including sex can enhance performance.

Kim et al. derived a model from the simplified Gullstrand eye, using *K*_mean_ and SER, with a correction constant for SER variation [14]. Their 382-participant Asian sample (ages 7–77 years) was measured without cycloplegia. They reported an AL overprediction of 0.18 ± 0.47 mm (95% LoA: –0.75 to +1.10 mm), with myopes showing the largest errors. In the present sample, overprediction pre-cycloplegia was higher (0.87 ± 0.60 mm; 95% LoA: –2.05 to +0.30 mm), especially in hyperopic and emmetropic eyes. Kim et al. [14] reported 75.5% and 95.5% of predictions falling within 0.5 mm and 1.0 mm, respectively—substantially higher than the present findings (23% ≤0.5 mm; 61% ≤1.0 mm). These differences likely reflect sample characteristics, since age and high ametropia reduce model precision [20].

Tang et al. developed an AL prediction model using linear regression and machine learning on 1011 myopic Asian children aged 6–18 years, obtained with cycloplegic refraction. Their predictors (*K*_mean_, SER, sex and age) explained 81% of AL variance—comparable to 82% (pre-cycloplegic) and 92% (post-cycloplegia) in the current study. The inclusion of age accounted for AL elongation during childhood and adolescence and the regression model was based on concurrent reduction in lens power [31–33].

Morgan et al. [13] proposed a linear regression model based on cycloplegic SER and *K*_mean_ in 144 Caucasian participants aged 8–12 years, later validated in 1046 individuals aged 6–22 years [13]. They reported an AL underestimation of 0.13 mm (95% LoA: –0.73 to +0.99 mm), consistent with the post-cycloplegic results of the present study (0.06 mm; 95% LoA: –0.72 to +0.84 mm). Queirós et al. [17] applied this model to 1783 participants aged 6–25 years (SER measured with an open-field autorefractor without cycloplegia) and observed an AL overestimation of 0.25 ± 0.48 mm (95% LoA: +0.70 to +1.20 mm), in line with the current pre-cycloplegia data [17]. Queirós et al. proposed a new model, adding age as a predictor, which explained ~80% of AL variability. In the present study, this model showed an 0.28 mm overprediction (one of the lowest) pre-cycloplegia and a 0.08 mm underprediction after cycloplegia—supporting the role of accommodation control in improving AL prediction.

Dutt et al. [18] developed a regression model using cycloplegic SER and *K*_mean_ from 1301 Caucasian adults aged 18–22 years. Under non-cycloplegic and cycloplegic conditions, their model overestimated AL by 0.10 ± 0.52 mm (95% LoA: –0.92 to +0.11 mm) and 0.01 ± 0.49 mm (95% LoA: –0.94 to +0.97 mm), respectively. In the present sample, overestimations were greater (pre-cycloplegia: –0.56 ± 0.55 mm; post-cycloplegia: –0.23 ± 0.38 mm), reaffirming that cycloplegia enhances precision and accuracy.

Lingham et al. [20] proposed a regression model based on cycloplegic SER, *K*_mean_, sex and age, based on data from 1068 Caucasians (6–20 years), 3429 Asians (5–18 years) and 240 Caucasian myopes (6–19 years). Their model underpredicted AL by 0.08 ± 0.40 mm (95% LoA: –0.71 to +0.87 mm) in a myopic cohort. In the present sample after cycloplegia, the model overpredicted AL slightly (–0.12 ± 0.35 mm; 95% LoA: –0.81 to +0.57 mm), likely due to the inclusion of hyperopic and emmetropic eyes.

Regression analysis of AL prediction errors against cycloplegic variations in *K*_mean_ and SER showed that ΔSER explained 97.3%–99.1% of the variance in predicted AL across all models, whereas mean corneal curvature (Δ*K*_mean_) had a minimal contribution (0.5%–1.2%).

Considering the reported repeatability of objective non-cycloplegic refraction (~0.65 D), objective cycloplegic refraction (~0.32 D) [14], and subjective refraction (~0.35 D) [34], the resulting AL prediction error may range from ~0.30 mm (non-cycloplegic) to ~0.15 mm (cycloplegic). These values are comparable with the errors observed in the present study and inherently limit the detection of subtle AL changes, confirming that the precision of SER measurement is the primary determinant of the accuracy and repeatability of AL prediction.

At this repeatability threshold, the models are inadequate to monitor physiological AL variation, which typically ranges from approximately 0.16 mm/year in younger children (6–9 years of age) to about 0.05 mm/year in adolescents older than 13 years [35]. While eyes exhibiting faster axial elongation (>0.20 mm/year) may allow detection of AL growth, the clinical and research utility of the AL prediction models remains limited for assessing the effectiveness of myopia control interventions, which aim to reduce AL elongation to approximately 0.10–0.25 mm/year [36], as changes that may fall within the intrinsic uncertainty of the prediction models. Nevertheless, despite these repeatability constraints, the AL prediction models remain clinically valuable as they provide a practical estimation of AL, enabling clinicians to stratify ocular risk and identify eyes potentially predisposed to pathological elongation when direct biometry is unavailable.

Where feasible, cycloplegic refraction improves the reliability of AL prediction models; however, its repeated use in paediatric populations requires careful consideration, as transient side effects may reduce patient tolerance and compliance with follow-up visits. When direct AL measurements are not readily available, combining periodic cycloplegic assessments with non-cycloplegic monitoring strategies may be a pragmatic approach in routine clinical practice.

A major strength of this study lies in the direct comparison of multiple AL prediction models on the same paediatric sample, both before and after cycloplegia. Since these models rely on routinely acquired clinical parameters, the findings offer practical insights into their clinical applicability. The inclusion of a balanced distribution of refractive error types allows for a broader and more representative comparison than the investigations of Tang et al. [16] and Lingham et al. [20], which were limited to myopic eyes. In the context of myopia progression, monitoring AL in emmetropic and low-hyperopic eyes is clinically relevant, as a reduction in hyperopia may indicate axial elongation [32].

Some limitations should be acknowledged. Analyses were restricted to the right eye only (one eye per participant); however, random eye selection has been recommended to minimise selection bias [37]. The sample size was smaller than those enrolled in the original model development studies. Nonetheless, this study aimed to compare rather than validate or train new models, for which the sample size was adequate.

Although inter-measurement repeatability was not formally assessed, each value obtained with the Myopia Master represents the average of three consecutive measurements. This reduces the impact of spurious readings and enhances measurement precision. Together with the documented high repeatability of this device, this supports the reliability of the measurements and the validity of the findings [38].

Additionally, only paediatric Caucasian participants were included, whereas most existing models were derived from Asian or mixed samples. Although ethnic differences in AL have been reported [39], they appear to stem mainly from ocular biometric relationships rather than ethnicity itself [20], supporting the validity of these comparisons while underscoring the need for broader cross-population assessments.

## Conclusions

This study highlights the importance of cycloplegic refraction for improving the accuracy and repeatability of AL predictive models. The models of Morgan et al. [13], Queirós et al. [17] and Lingham et al. [20] demonstrated minimal bias and superior repeatability under cycloplegic conditions. SER was the primary factor influencing prediction errors, while corneal curvature had a negligible impact. Overall, AL predictions were more accurate in myopic than in hyperopic or emmetropic eyes, where mild overprediction persisted. These models provide a useful alternative in primary care settings without optical biometers, particularly for paediatric myopia management, though they should not replace direct AL measurements when available.

## Data Availability

No datasets were generated or analysed during the current study.
